# A rapid approach for discriminating *Ganoderma* species using attenuated total reflectance–Fourier transform infrared (ATR-FTIR) spectroscopy integrated with chemometric analysis and convolutional neural network (CNN)

**DOI:** 10.3389/fchem.2025.1655760

**Published:** 2025-10-27

**Authors:** Sze Yun Chen, Chi Yuan Low, Jun Yang Loh, Wan Yin Tew, Li Yun Ouyang, Peng Shun Ong, Chong Seng Yan, Hui Wei Loh, Ying Chen, Wei Xu, Wen Xu, Tiem Leong Yoon, Mun Fei Yam

**Affiliations:** 1 Collage of Pharmacy, Fujian University of Traditional Chinese Medicine, Fuzhou, Fujian, China; 2 School of Pharmaceutical Sciences, Universiti Sains Malaysia, Minden, Malaysia; 3 School of Physics, Universiti Sains Malaysia, Minden, Malaysia

**Keywords:** ATR-FTIR, chemometric analysis, CNN, *Ganoderma lucidum*, *Ganoderma sinense*, *Ganoderma tsugae*, Ling Zhi

## Abstract

The issue of adulteration and misclassification of *Ganoderma* species is addressed in this research. In the study, we present a novel and comprehensive framework for *Ganoderma* authentication by analyzing attenuated total reflectance–Fourier transform infrared (ATR-FTIR) spectra using a combined approach of a chemometric analysis and deep learning (DL) with a convolutional neural network (CNN). The three *Ganoderma* species involved in this study were as follows: *Ganoderma lucidum*, *Ganoderma sinense*, and *Ganoderma tsugae*. Among chemometric models, orthogonal partial least squares discriminant analysis (OPLS-DA) yielded a high accuracy of 98.61%, a sensitivity of 97.92%, and a specificity of 98.96%. Additionally, the root-mean-squared error of estimation (RMSEE), root-mean-squared error of prediction (RMSEP), and root-mean-squared error of cross-validation (RMSECV) values for the OPLS-DA model were <0.3, confirming its reliability. The CNN model also performed well, achieving 89.84% accuracy, 84.75% sensitivity, and 92.38% specificity, with minimal variation during random segregation testing. Additionally, the model exhibited a precision of 0.87 ± 0.02, a recall of 0.85 ± 0.03, and an F1 score of 0.86 ± 0.03 for 10 random segregation tests. As a conclusion, both chemometric and CNN models developed in this study are efficient and robust for classifying *Ganoderma* species. To further validate this combined approach, we aim to implement chemometric and CNN models in other medicinal herb authentication in the future.

## 1 Introduction

*Ganoderma* (Ling Zhi) is a traditional medicinal mushroom from the *Ganodermataceae* family, used in Chinese and Asian communities. Its earliest record appears in Shen Nong Ben Cao Jing (ca. 100 B.C.), and it has been mentioned in many ancient texts. *Ganoderma* can now be cultivated on a large scale for research and medicinal purposes. More than 20 species have been studied, but *Ganoderma lucidum* (red Ling Zhi) is the most researched, whereas *Ganoderma sinense* (purple Ling Zhi) is also important in China. Since 2001, *Ganoderma tsugae* has been approved for use in health products. These three species are officially listed by the Chinese government as suitable for use in health foods.

The health benefits of Ling Zhi are primarily attributed to its active compounds, such as triterpenes and polysaccharides. Triterpenes exhibit strong pharmacological effects, including antitumor, liver protection, anti-angiogenic, and antihistaminic activities, whereas polysaccharides enhance immune function (Jong and Birmingham, 1992; Su et al., 1999; Zhang et al., 2019). *Ganoderma lucidum* has been used in the treatment of conditions such as neurosis, polymyositis, dermatomyositis, atrophic myotonia, and muscular dystrophy (Zhang et al., 2019). *Ganoderma sinense* is often used as a supportive treatment for leukopenia and bone marrow damage caused by chemotherapy or radiotherapy (Zhang et al., 2019). Due to its medicinal value, Ling Zhi extracts are widely commercialized as health foods, such as drinks, coffee powders, supplements, and syrups (Lai et al., 2004).

Due to the interest in high economic gains and the intra-species similarity, fraudulent inclusion of adulterated Ling Zhi occurs in the market, impacting the authenticity of Ling Zhi products (Fu et al., 2017; Lin and Yang, 2019; Wachtel-Galor et al., 2011). The adulteration of *Ganoderma* species is also attributed to the heightened demand due to its diverse benefits as a functional food. The imperilment of herbal product security, the challenge to authority-managing institutions, and the erosion of consumer trust in Ling Zhi products collectively emphasize the need for an approach to accurately identify *Ganoderma* species and distinguish them from adulterants.

In the authentication of *Ganoderma* species, there are several discrimination methods, such as DNA barcoding, high-performance liquid chromatography (HPLC), thin-layer chromatography (TLC), and capillary electrophoresis (Loyd et al., 2018; Sheng et al., 2022; Sun et al., 2014; Yao et al., 2021). However, these methods are not considered in this research because they require extensive sample preparation and a time-consuming procedure. To meet the efficiency demands of the pharmaceutical industry and regulatory bodies in large-scale herbal authentication, spectroscopic methods that are rapid, simple, and nondestructive can be the best alternative to replace traditional methods (Al-Hetlani et al., 2025). Spectroscopic methods such as attenuated total reflectance–Fourier transform infrared (ATR-FTIR) spectroscopy, near-infrared (NIR) spectroscopy, and Raman spectroscopy offer minimal sample preparation, providing a strong molecular fingerprint for the authentication of *Ganoderma* species (Al-Hetlani et al., 2025; Amin et al., 2021; Chen et al., 2008; Wang et al., 2019). Although NIR spectroscopy is rapid and nondestructive, its broad overtone and combination bands (12,500 to 4,000 cm^−1^) provide less clear molecular information, limiting its ability to distinguish closely related herbal species (Wang and Yu, 2015). Raman spectroscopy is affected by fluorescence interferences commonly observed in plant matrices, which can obscure important spectral features (Al-Hetlani et al., 2025). In contrast, ATR-FTIR spectroscopy is preferred for precise authentication because it has superior functional group resolution and robustness against fluorescence interferences.

ATR-FTIR spectroscopy is a rapid, nondestructive, and cost-effective technique that captures molecular vibrational information from samples with minimal preparation (Tiernan et al., 2020). To interpret its complex spectra, chemometric methods such as principal component analysis (PCA), PCA-Class, and orthogonal partial least squares discriminant analysis (OPLS-DA) are commonly applied to reduce dimensionality, identify patterns, and discriminate between groups (Tew et al., 2022). More recently, the convolutional neural network (CNN) has emerged as a promising approach as they can automatically learn features directly from data without manual extraction (Nichols et al., 2019). Although chemometric models remain powerful, the CNN offers scalability and adaptability, making it a valuable complementary tool for herbal authentication (Li et al., 2022).

Before our work, there was research focused on the discrimination of *Ganoderma* species using ATR-FTIR spectroscopy combined with a chemometric approach (Wang et al., 2019). Nevertheless, they did not discover the use of deep learning (DL) to discriminate *Ganoderma* species. Acknowledging the influential studies of Wang et al. (2019), this research extends and refines the method for discriminating *Ganoderma* species using ATR-FTIR spectroscopy combined with chemometric methods, incorporating the CNN. In recent years, DL has become increasingly utilized in the field of automatic identification of crop diseases, plant phenotyping, and plant species classification via leaf classification (Boulent et al., 2019; Jiang and Li, 2020; Lee et al., 2023). A literature review conducted prior to this study proposed limited data resources on the classification of *Ganoderma* species using the CNN. In this research, a deep learning method through the CNN, together with a chemometric analysis, is explored for better identification of *Ganoderma* species.

## 2 Methodology

### 2.1 Samples and materials

In this study, 118 *Ganoderma* samples were used, which were contributed by Fujian University of Traditional Chinese Medicine. The sample set comprises three distinct *Ganoderma* species, namely, *G. lucidum* (Leyss.ex Fr.) P. Karst (GL), *G. sinense* Zhao. Xu et Zhang (GS), and *G. tsugae* Murrill (GT). In the sample set, there are 78 GL, 20 GS, and 20 GT. These samples were sourced from China. The samples were dried in an oven at 50 °C for 8–9 h. Afterward, the samples were ground into a fine powder, sieved through a 200-mesh stainless steel sieve, and then stored at 8 °C before the experiment. Prior to the ATR-FTIR analysis, the samples were reheated at 50 °C for an hour.

### 2.2 Data acquisition and processing

A Spectrum Two™ FTIR Spectrometer (PerkinElmer, United States), equipped with a Universal Attenuated Total Reflectance (UATR) accessory, was used in this study. A sufficient amount of the powdered *Ganoderma* sample was placed to fully cover the UATR crystal surface. A consistent pressure was applied to all samples to ensure consistent contact on the crystal surface. The infrared spectra measurement step was performed in a room with controlled humidity and temperature to minimize the impact of environmental factors on the measured spectra. The spectra were then recorded as 36 scans in the wavelength ranging from 4,000 to 400 cm^−1^, with a resolution of 4 cm^−1^ and an interval of 1 cm^−1^, to improve the signal-to-noise ratio and spectral resolution. Software Spectrum 10.5.3 (PerkinElmer, United States) was utilized to analyze the spectra of samples in this research.

ATR correction was performed to mathematically correct the measured ATR-FTIR spectra, compensating for the attenuation of infrared radiation (Beasley et al., 2014). The baselines of the spectra were corrected. A smoothing step was performed to reduce the noise in the spectra. An arithmetic operation involving a subtraction manipulation step was carried out in conjunction with normalization for the baseline and smoothed spectra.

### 2.3 Data analysis

#### 2.3.1 Chemometric analysis

An unsupervised pattern recognition technique known as the PCA was implemented to determine differences in ATR-FTIR spectral characteristics among the *Ganoderma* species. A discrimination study was then conducted after the PCA, in which PCA-class and OPLS-DA were performed. The samples were randomly divided into two sets: one for calibration purposes and the other for validation. These sets were utilized to implement the PCA-class and OPLS-DA model. The calibration set comprises 60% of the spectra from the three different *Ganoderma* species, with the validation set containing the remaining 40%. Internal validation was performed using a permutation test, which consisted of 100 permutations. The chemometric analysis was done using SIMCA version 14.1 (Umetrics, Sweden). The accuracy, sensitivity, and specificity were calculated using Formulas 1–3, respectively:

$$Accuracy=\frac{TP+TN}{TP+TF+FP+FN}\times 100\%,$$ (1)

$$Sensitivity=\frac{TP}{TP+FN}\times 100\%,$$ (2)

$$Specificity=\frac{TN}{TP+FP}\times 100\%,$$ (3)

where

TP = true positive

TN = true negative

FP = false positive

FN = false negative

#### 2.3.2 Convolutional neural network

Although a total of 118 *Ganoderma* samples were not too few for chemometric analysis, this quantity of samples available for the classification of *Ganoderma* species using the CNN was deemed insufficient. This is due to the rarity and high cost of obtaining *Ganoderma* samples. Apart from having a limited number of samples, the scarcity of sample sources also leads to a significant imbalance across different species of *Ganoderma*. In particular, both GS (20 samples) and GT (20 samples) were underrepresented compared to GL (78 samples), which had a considerably larger number of samples. This can lead to class imbalance, where underrepresented classes are poorly identified compared to their well-represented counterparts. Apart from that, the accuracy of the model could be greatly affected or lead to a misleadingly high overall accuracy score (Venkataramana et al., 2022).

The dataset for this study comprised 1-dimensional ATR-FTIR spectral curves (coefficient vs wavenumber) obtained from GL, GS, and GT. The complete dataset consisted of 118 samples, with an uneven distribution across classes, as detailed in Table 1.

**TABLE 1:** Breakdown of *Ganoderma* samples used in this study.

Class	Raw sample count	Raw sample count in group A	Raw sample count in group B	Oversampled sample count in group A	Oversampled sample count in group B
*Ganoderma lucidum*	78	39	39	500	500
*Ganoderma sinense*	20	10	10	500	500
*Ganoderma tsugae*	20	10	10	500	500
Total	118	59	59	1,500	1,500

##### 2.3.2.1 Data augmentation

Data augmentation was performed on both the training and testing sets to address class imbalance, which can lead to biased outcomes in machine learning models. In this research, the Synthetic Minority Oversampling Technique (SMOTE) algorithm, the data augmentation package, was applied to both training and testing sets. The SMOTE generates synthetic samples based on the existing samples provided by comparing randomly selected data points in a minority class (i.e., GS and GT) with their closest neighboring data point and generating new data along the lines of these neighbors (Venkataramana et al., 2022). Typically, the SMOTE uses five nearest neighbors (Elreedy and Atiya, 2019).

The original dataset consisted of three classes: 78 GL, 20 GS, and 20 GT. Each class was randomly split into two mutually exclusive subsets, namely, group A and group B. Both groups contained as equal a number of samples per class as possible. No sample overlapped between group A and group B. Subsequently, the SMOTE was used to oversample each class in both groups independently, resulting in 500 samples, which are presented in Table 1.

##### 2.3.2.2 Training and testing phases

The dataset in group A was used to train a CNN-based classifier. During training, cross-validation was implemented as an intermediate evaluation step to optimize hyperparameters and improve model generalization, thus reducing the risk of overfitting. In particular, stratified k-fold cross-validation was applied to equally partition the dataset into multiple folds. In each iteration, the CNN model was trained on a subset of the data and validated on a separate fold. This process was repeated for all folds, and the resulting performance metrics were averaged to obtain a reliable estimate of the model’s generalization performance. The implementation was carried out using the standard Keras API integrated with scikit-learn’s cross-validation tools to ensure seamless and reproducible model training and evaluation. In this study, the *n_splits* parameter in the KFold function in scikit-learn was set to 10, which resulted in the dataset being equally partitioned into 10 folds.

The CNN is inherently stochastic, indicating that even when using the same architecture and training dataset, slight variations in model performance can occur across different training runs. In practical applications, model evaluation involves training the CNN on one portion of the data and assessing its predictive performance on a separate, unseen portion to estimate its generalization capability. In this study, the dataset from group B was used as an independent testing set to evaluate the performance of the trained CNN on group A. The output of the testing phase was a confusion matrix, which would be used to evaluate the model’s performance in this phase. Key metrics, including precision, recall, and F1 score, were recorded during this testing phase.

##### 2.3.2.3 Random segregation test

A random segregation test was used to assess the robustness and consistency of performance across different runs. If the model demonstrates high and consistent accuracy across different runs, it indicates that the CNN is robust and not overly dependent on specific data samples. However, a large fluctuation in accuracy would indicate that the model’s performance is unstable. In this study, the random segregation test was performed 10 times, where the testing dataset was tested with 10 folds of the trained dataset. The confusion matrix of each random segregation test was recorded. The accuracy, sensitivity, and specificity for each random segregation test were calculated. The mean, standard deviation, and coefficient of variation (CV) for accuracy, sensitivity, specificity, precision, recall, and F1 score were calculated using Statistical Package for the Social Sciences (SPSS) version 27 software (IBM Corp, United States) for 10 random segregation tests.

##### 2.3.2.4 Architecture of the CNN model

**FIGURE 1:**
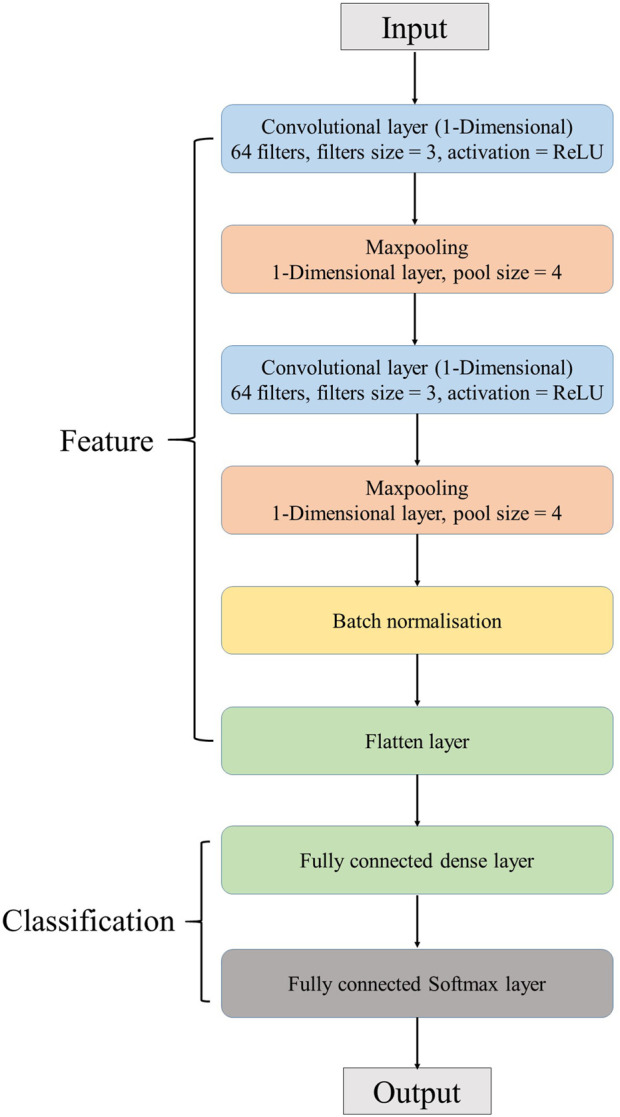
Architecture of the CNN model.

The deep learning approach in this research applied a CNN model as the machine learning model. The architecture of the CNN model in this study was designed to be relatively straightforward. Simply, it was designed to consist of two convolutional layers, two max pooling layers, and two dense activation layers. The details and features of the CNN model used in this research are discussed in the Supplementary Material. Figure 1 presents the architecture of the CNN model in this research.

## 3 Result and discussion

### 3.1 Differentiation using ATR-FTIR spectra

**FIGURE 2:**
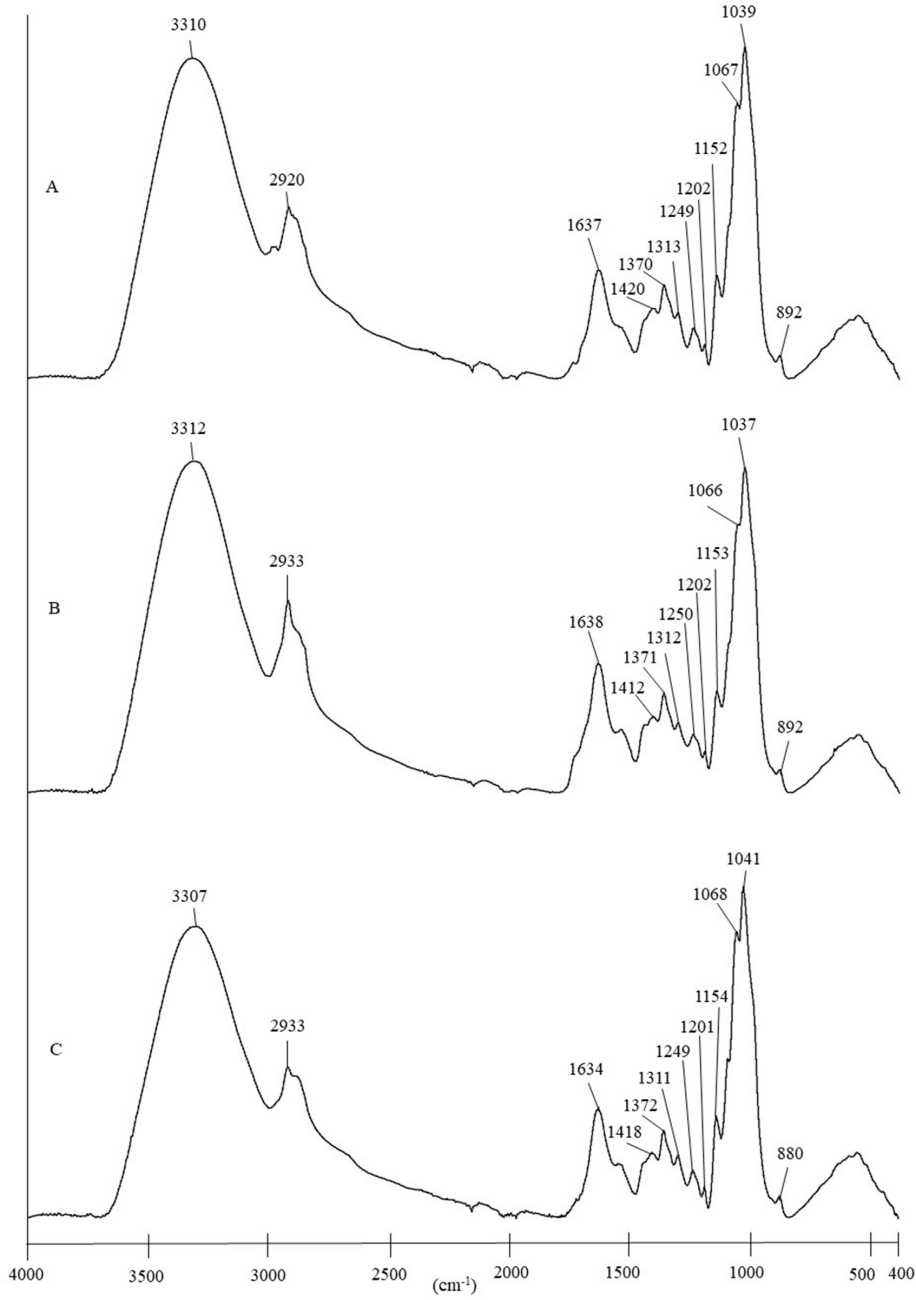
Comparison of ATR-FTIR spectra of **(A)** *Ganoderma lucidum*, **(B)** *Ganoderma sinense*, and **(C)** *Ganoderma tsugae*.

ATR-FTIR spectral profiles for the various *Ganoderma* species are presented in Figure 2. No significant variance was observed in the ATR-FTIR spectra of the three species. This indicated that the functional groups or bioactive components in each *Ganoderma* species were almost similar. The assignments of the absorption bands and their possible compounds are presented in Table 2.

**TABLE 2:** Peak assignment of ATR-FTIR spectra of three *Ganoderma* species.

Peak	(cm^-1^)	*G. tsugae*	Primary assignment	Functional group	Biomolecule
*G. lucidum*	*G. sinense*				
3310	3312	3307	O − H, ν	Alcohols/phenols	Triterpenes and polysaccharides
2,920	2,923	2,923	C − H, ν _as_ O − H, ν	Alkane	Various
1,637	1,638	1,634	C=O, ν N − H, δ	Amide I	Protein
1,420	1,412	1,418	=CH, δ _ip_	Alkene	Various
1,370	1,371	1,372	C − H, δ	Alkane	Various
1,313	1,312	1,311	C − N, ν N − H, δ	Amide III	Protein
1,249	1,250	1,249	C − N, ν N − H, δ	Amide III	Protein
1,202	1,202	1,201	C − N, ν N − H, δ	Amide III	Protein
1,152	1,153	1,154	C − O, ν	Ketone	Saccharides/glycosides
1,067	1,066	1,068	C − O, ν	Ketone	Saccharides/glycosides
1,039	1,037	1,041	C − O, ν	Ketone	Saccharides/glycosides
892	892	880	=CH, δ _oop_	Alkene	Saccharides/glycosides

As tabulated in Table 2, the common absorption bands observed in the ATR-FTIR spectra of *Ganoderma* species are at 3,310 cm^−1^, 2,920 cm^−1^, 1,637 cm^−1^, 1,420 cm^−1^, 1,370 cm^−1^, 1,313 cm^−1^, 1,249 cm^−1^, 1,202 cm^−1^, 1,152 cm^−1^, 1,067 cm^−1^, 1,039 cm^−1^, and 892 cm^−1^. A strong band observed at approximately 3,310 cm^−1^ was attributed to O–H stretching of polysaccharides and triterpenes (Sun et al., 2011; Wang et al., 2019). This finding aligns with the fact that *Ganoderma* triterpenes and polysaccharides are significant biomolecules or active ingredients in *Ganoderma* species, contributing to a wide range of therapeutic characteristics, including anti-inflammatory and antitumor activities (Lin and Yang, 2019; Xia et al., 2014). Next, the presence of an absorption band representing amide I at approximately 1,630 to 1,645 cm^−1^ was attributed to partially denatured collagen (Kristoffersen et al., 2023). The band observed in the region between approximately 1,200 and 1,315 cm^−1^ arises from the stretching vibrations of the C–N bond and bending vibrations of the N–H bond of amide III proteins (Sun et al., 2011). Absorption bands near 1,152 cm^−1^, 1,067 cm^−1^, 1,039 cm^−1^, and 892 cm^−1^ also indicate the presence of polysaccharides as another significant active ingredient in *Ganoderma* species. Among these bands, those at approximately 1,067 cm^−1^ and 1,039 cm^−1^ were relatively strong. Another small band found at approximately 892 cm^−1^ signified the presence of the β-anomer configuration of the saccharides (Lin and Yang, 2019).

### 3.2 Differentiation through chemometric analysis

**FIGURE 3:**
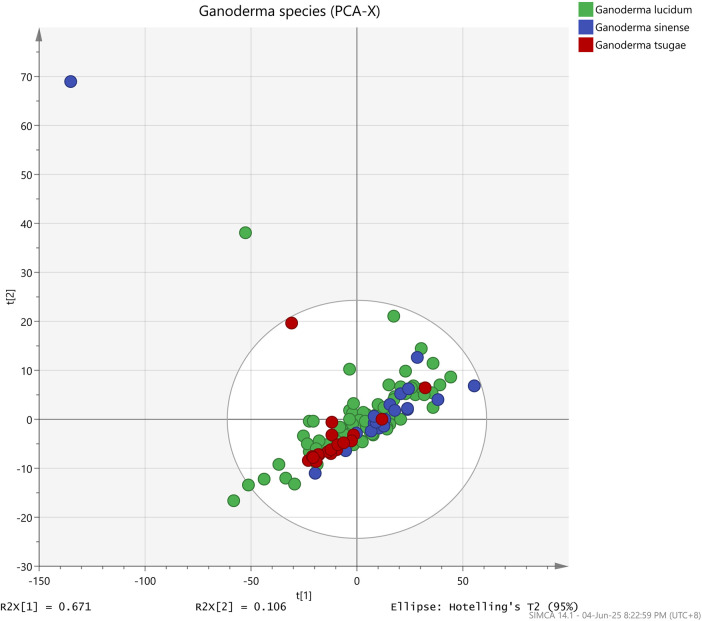
Unsupervised PCA score plot of three different *Ganoderma* species: (green) *Ganoderma lucidum*, (blue) *Ganoderma sinense*, and (yellow) *Ganoderma tsugae*.

In this study, we applied the unsupervised PCA, supervised PCA-Class, and OPLS-DA methodologies to visually present and distinguish among the three species of *Ganoderma* samples obtained from the ATR-FTIR analysis. In the PCA, R^2^X and Q^2^ were commonly used to assess the model’s performance and reliability, respectively. R^2^X is the proportion of total variance in the independent variables (X) that is captured by the principal components, whereas Q^2^ reflects the model’s predictive capability. Values of R^2^X and Q^2^ close to 1 indicate that the model has high reliability and strong predictive power (Kotzé-Hörstmann et al., 2022; Liu et al., 2018). The score plot depicted in Figure 3 illustrates significant variability among all samples in the PCA, as evidenced by R^2^X = 0.99 and Q^2^ = 0.98. However, the PCA model proved inadequate in distinguishing between the various *Ganoderma* species effectively.

In contrast, the supervised PCA-class model demonstrated R^2^X values between 0.99 and 1.00 and Q^2^ values between 0.94 and 0.98 for the three *Ganoderma* species. The PCA-class model achieved an accuracy of 98.31%, a sensitivity of 97.46%, and a specificity of 98.73%. From these results, we can conclude that the PCA-class model is capable for classifying the *Ganoderma* species. The results are encouraging although there remains potential for further improvement.

**FIGURE 4:**
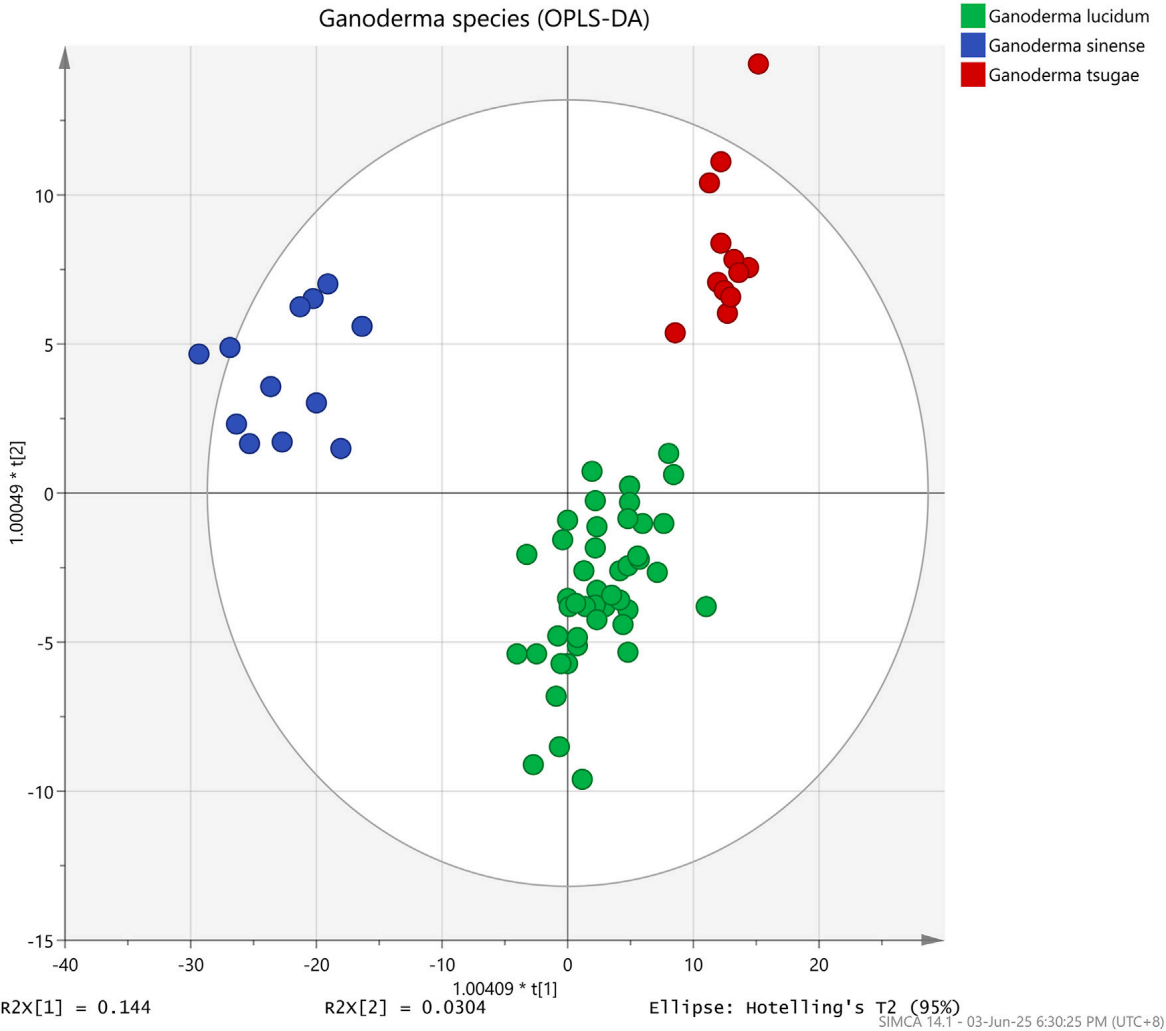
Supervised OPLS-DA score plot of three different *Ganoderma* species: (green) *Ganoderma lucidum*, (blue) *Ganoderma sinense*, and (yellow) *Ganoderma tsugae*.

**FIGURE 5:**
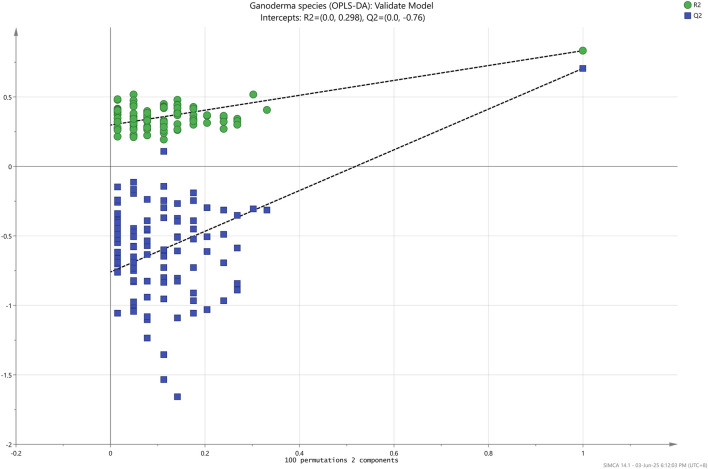
Permutation test of the OPLS-DA model.

Nevertheless, the data were successfully differentiated into three different species (GL, GS, and GT) when implementing an orthogonal algorithm in the OPLS-DA analysis, as shown in Figure 4. R^2^X, the goodness of fit parameter (R^2^Y), and the goodness of prediction parameter (Q^2^Y) were scrutinized to evaluate the overall efficacy of the OPLS-DA prediction model. The values of these parameters fall within 0–1, with values approximately 1 indicating excellent predictive capability and values between 0.50 and 0.90 denoting good predictive capability (Jingying et al., 2023; Tew et al., 2022). As detailed in Table 3, the OPLS-DA model in this research was deemed as a good predictive model as R^2^X, R^2^Y, and Q^2^Y values were 0.99, 0.85, and 0.72, respectively. For the permutation test, R^2^Y and Q^2^Y intercepts were 0.30 and −0.76, respectively, as depicted in Figure 5, indicating that the model fits one another. Furthermore, the root-mean-squared error of estimation (RMSEE), root-mean-squared error of prediction (RMSEP), and root-mean-squared error of cross-validation (RMSECV) were evaluated to assess the accuracy and predictability of the OPLS-DA model. The values ranged from 0 to 1, where the smaller the value of RMSEE and RMSEP, the better the predictability and accuracy of the model (van Wyngaard et al., 2021). Meanwhile, for RMSECV, the smaller the value, the less the variable, including noise removal (Takahama and Dillner, 2015). According to Table 3, the values of RMSEE (0.21), RMSECV (0.26), and RMSEP (0.25) were considered small, affirming the suitability and accuracy of the OPLS-DA model. Additionally, the OPLS-DA model demonstrated 98.61% accuracy, 97.92% sensitivity, and 98.96% specificity in classifying *Ganoderma* species, making it an ideal methodology for achieving the objectives outlined in this experiment.

**TABLE 3:** Parameters of PCA, PCA-class, and OPLS-DA models.

Species	R^2^X	R^2^Y	Q^2^Y	Q^2^	R^2^Y intercept	Q^2^Y intercept	RMSEE	RMSECV	RMSEP	Accuracy	Sensitivity	Specificity
PCA	0.99	—	—	0.98	—	—	—	—	—	—	—	—
PCA-class												
G. *lucidum*	0.99	-	-	0.98	—	—	—	—	—	98.31%	97.46%	98.73%
G. *sinense*	1.00	-	-	0.96	—	—	—	—	—			
G. *tsugae*	0.99	-	-	0.94	—	—	—	—	—			
OPLS-DA	0.99	0.85	0.72	-	0.30	−0.76	0.21	0.26	0.25	98.61%	97.92%	98.96%

### 3.3 Differentiation using the CNN

#### 3.3.1 Performance evaluation of the training phase

**FIGURE 6:**
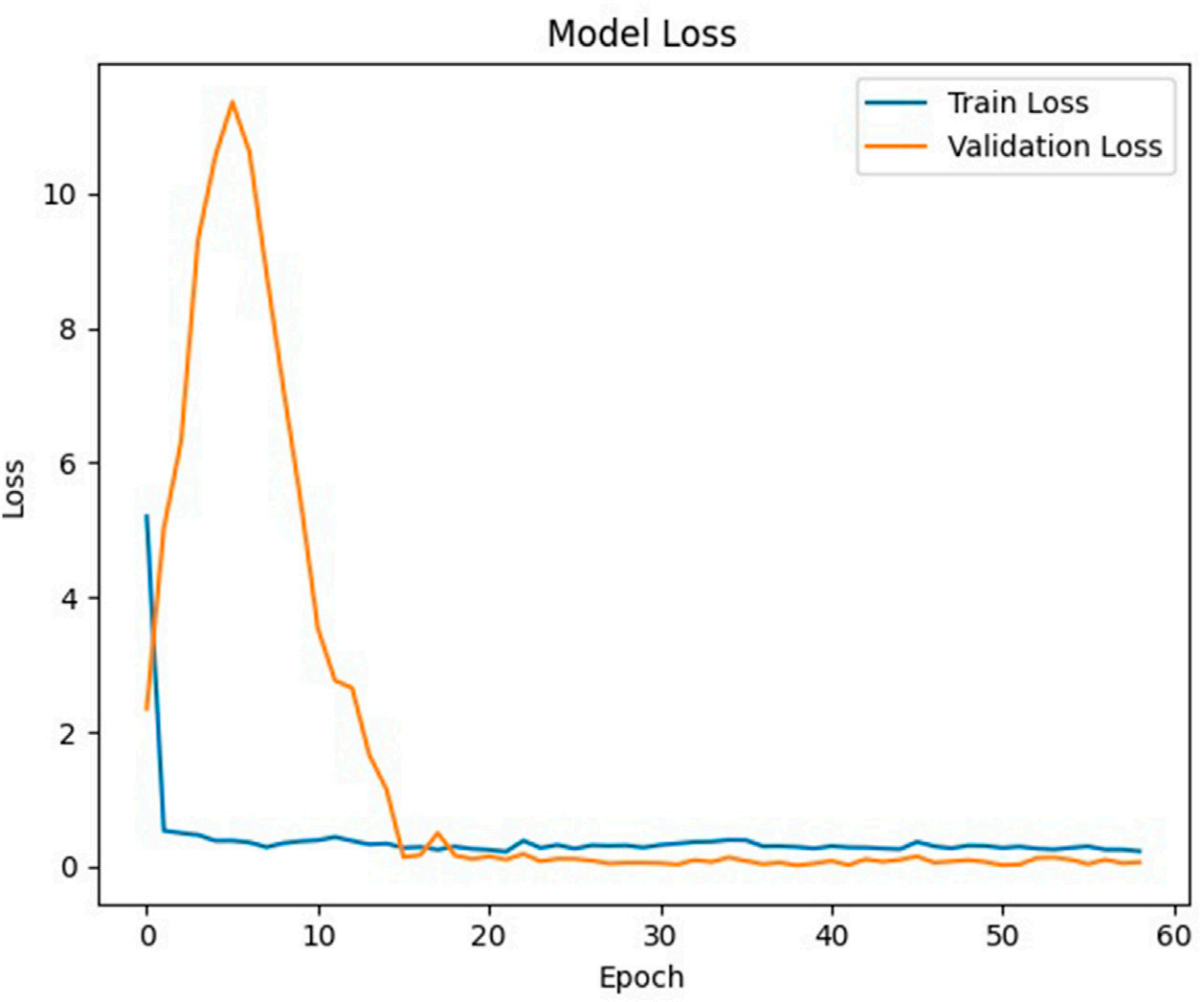
Learning curve of the training and validation loss of the CNN model.

The performance of the CNN model was evaluated at the end of each epoch. The standard practice is to plot and evaluate the learning curves of the model at the training stage. Consequently, we can access the model fit of the CNN model. Model fit issues, such as overfitting and underfitting, are common problems in machine learning, which could lead to poor performance and low accuracy in the model. Overfitting is represented by a convergence and subsequent divergence of the two plots in the learning curves. Additionally, we can identify underfitting by examining a noticeable gap between the training and validation loss curves in the learning curves. To prevent overfitting, the general guideline is to stop further training when the training loss levels off. Figure 6 shows one of the learning curves of the training and validation loss of a CNN model during the training process in this study. The learning curve is well fitted, with both curves converging at the same points. Additionally, the training and validation loss curves level off at nearly the same values, indicating that the model accurately predicts the samples without overfitting.

#### 3.3.2 Performance evaluation of random segregation tests

The robustness of our model in classifying *Ganoderma* fingerprints was assessed by observing the fluctuations in accuracy, sensitivity, and specificity across each random segregation test. A robust and reliable CNN model is expected to deliver consistent and stable performance, even with individual random segregations, without excessive statistical fluctuations. In cases where the model lacks robustness, the potential anticipating factor may stem from the dataset. For instance, an imbalanced dataset, a mishandled dataset, and a dataset with underrepresented samples could cause fluctuations in performance and deteriorate the model’s robustness.

In the context of this research, as referred to Table 1, the samples in the initial raw dataset were imbalanced. In contrast, the augmented dataset had a relatively higher number of samples overall, with each class of *Ganoderma* species having an equal number of samples, resulting in a balanced distribution. The random segregation tests were initially conducted using the initial raw dataset. However, the data were not reported in this study. To conclude, both underrepresented classes, GS and GT, showed significant fluctuations in the accuracy, sensitivity, and specificity across each repetition. Conversely, the fluctuation in GL with a higher number of samples was smaller. These outcomes further supported that the initial raw dataset was insufficient to train a robust CNN classifier with reliable performance. Additionally, this demonstrates the importance of the data augmentation step in yielding a robust CNN classifier.

**FIGURE 7:**
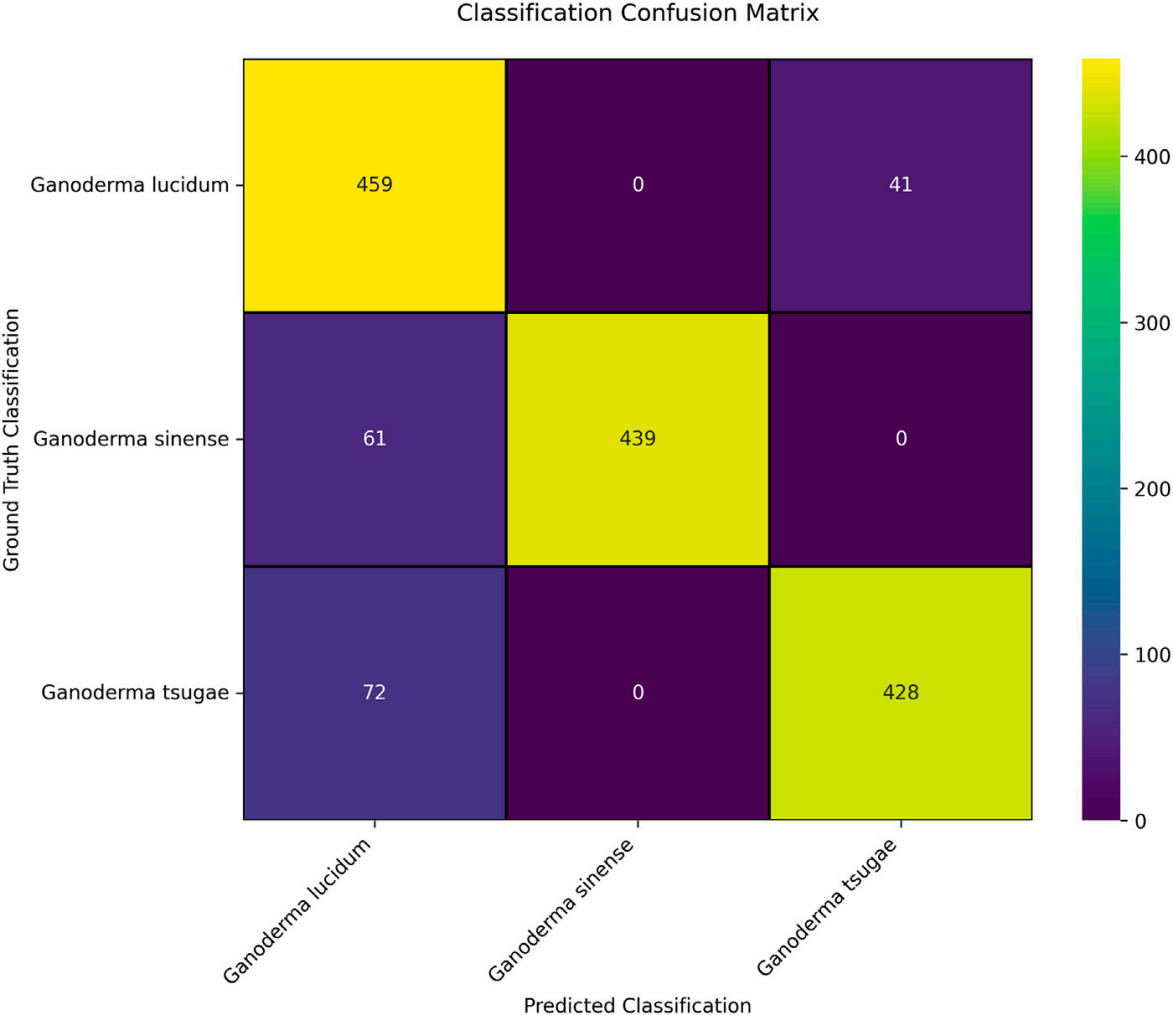
Confusion matrix of the random segregation test of the CNN model.

Subsequently, the random segregation test was then conducted using the oversampled dataset. The fluctuation in performance for each random segregation tests was observed and recorded using a multiclass confusion matrix function provided by scikit-learn in Python. A multi-class confusion matrix is commonly used to evaluate the performance of a CNN classifier as it is resilient toward various types of data distribution and data relationship (Ruuska et al., 2018). From the confusion matrix, information on how the *Ganoderma* species is correctly predicted or classified can be gathered. From the row of the confusion matrix, we can obtain the predicted values; conversely, we can obtain the true values from the column. In Figure 7, a confusion matrix from one of the random segregation tests is presented. From the confusion matrices of 10 random segregation tests, we observed that the classification of GL was better than that of GS and GT. Additionally, we observed that most of the misclassified GLs belong to the GT group, whereas most of the misclassified GSs and GTs belong to the GL group.

According to Table 4, the CNN model exhibited 89.84% ± 1.96% accuracy, 84.75% ± 2.95% sensitivity, and 92.38% ± 1.47% specificity in 10 random segregation tests. Additionally, the model exhibited a precision of 0.87 ± 0.02, a recall of 0.85 ± 0.03, and an F1 score of 0.86 ± 0.03 for 10 random segregation tests. The F1 score was calculated based on the harmonic mean of precision and recall, where values closer to 1 indicate that precision and recall are less deviant from each other, suggesting better performance in classifying predictions into the correct class (Hand et al., 2021). Hence, the F1 score value of 0.86 indicated good performance in the classification model. In addition, the CNN model demonstrated robustness, as indicated by the low CV for accuracy (2.18%), sensitivity (3.48%), specificity (1.59%), precision (2.30%), recall (3.55%), and F1 score (2.96%), reflecting minimal variability in performance metrics across 10 random segregation tests. Although the performance of the CNN in terms of accuracy, sensitivity, and specificity did not surpass that of OPLS-DA in the chemometric analysis, it still yielded satisfactory results.

**TABLE 4:** Summary of the results for random segregation tests.

Random segregation test	Accuracy (%)	Sensitivity (%)	Specificity (%)	Precision	Recall	F1 score
1	88.98	83.47	91.73	0.86	0.84	0.85
2	91.82	87.73	93.87	0.90	0.88	0.89
3	85.16	77.73	88.87	0.83	0.78	0.80
4	90.67	86.00	93.00	0.88	0.86	0.87
5	91.24	86.87	93.43	0.89	0.87	0.88
6	90.67	86.00	93.00	0.88	0.86	0.87
7	88.22	82.33	91.17	0.86	0.82	0.84
8	89.78	84.67	92.33	0.87	0.85	0.86
9	89.56	84.33	92.17	0.87	0.84	0.86
10	92.27	88.40	94.20	0.90	0.89	0.89
Mean ± standard deviation	89.84 ± 1.96	84.75 ± 2.95	92.38 ± 1.47	0.87 ± 0.02	0.85 ± 0.03	0.86 ± 0.03
CV	2.18%	3.48%	1.59%	2.30%	3.55%	2.96%

^*^Accuracy, sensitivity, and specificity were calculated from each confusion matrix. Precision, recall, and F1 score were summarized from each *Ganoderma* species based on its corresponding confusion matrix separately.

### 3.4 Comparison of the chemometric analysis and CNN

The chemometric analysis and CNN have their own strengths and limitations. First, in terms of data interpretability, chemometric methods are generally more straightforward and easier to comprehend (Omar et al., 2019). These approaches enable clear analysis and concise interpretation of relationships between input variables. In contrast, the CNN operates through more complex mechanisms, making its outputs less intuitive and more challenging to interpret (Liu et al., 2021). Chemometric techniques are typically preferred for preliminary analyses involving smaller and less complex datasets. Although modern chemometric tools, including multivariate analysis, classification, and prediction techniques, can enhance model performance, they may still face limitations when dealing with large-scale, nonlinear, and complex datasets (Kharbach et al., 2023). On the other hand, the CNN is well suited for handling high-dimensional and more intricate data structures (Zhu et al., 2023). Their layered architecture enables them to automatically extract relevant features during training, contributing to the development of a robust model (Debus et al., 2021). In addition, the inclusion of the CNN adds an innovative, automated feature extraction capability, minimizing the reliance on spectral preprocessing. Although the performance of the CNN was slightly lower than that of the chemometric analysis, its advantages highlight the need to consider the CNN as a valuable approach for discriminating *Ganoderma* species. In conclusion, the combined use of the chemometric analysis and CNN offers a strategic advantage by harnessing the strengths of each approach to offset their individual limitations, ultimately contributing to the development of a more reliable and effective classification model.

## 4 Conclusion

The classification and discrimination of *Ganoderma* species are areas in which this research is of particular importance as it not only refines the current understanding of ATR-FTIR spectroscopy combined with chemometric methods for classifying visually similar *Ganoderma* samples but also uses a deep learning approach to predict the class of different *Ganoderma* species. The findings on the conventional ATR-FTIR spectra of three *Ganoderma* species in this research further validate that the subtle variance in spectra, which are imperceptible to the naked eye, necessitates sophisticated analytical methods for accurate classification. The chemometric approach, such as OPLS-DA, applied in this research, has been proven to be a promising approach for discriminating *Ganoderma* species. Ultimately, this research has demonstrated that the CNN model can be a reliable approach for the discrimination between different *Ganoderma* species. Although the CNN model achieved slightly poorer performance, it offers significant advantages in scalability, adaptability to larger and more complex datasets, and the potential for real-time implementation in routine authentication workflows. The combined approach advances the field by bridging the traditional chemometric rigor with modern deep learning flexibility, thereby opening new avenues for robust, rapid, and nondestructive authentication of medicinal fungi. Looking forward, this approach may be extended to other medicinal herbs, with the ultimate goal of developing a publicly accessible authentication platform to support research, industry, and regulatory applications. Although the model’s performance has been proven in this study, it remains essential to validate it in other medicinal herb authentication to identify areas for improvement and implement necessary upgrades accordingly.

## Data Availability

The original contributions presented in the study are included in the article/Supplementary Material; further inquiries can be directed to the corresponding authors.
