# Supplementary figures and images for: A rapid approach for discriminating Ganoderma species using attenuated total reflectance–Fourier transform infrared (ATR-FTIR) spectroscopy integrated with chemometric analysis and convolutional neural network (CNN)
Source: Front Chem. 2025 Oct 27;13:1655760. doi: 10.3389/fchem.2025.1655760 (PMC12597922; doi:10.3389/fchem.2025.1655760)

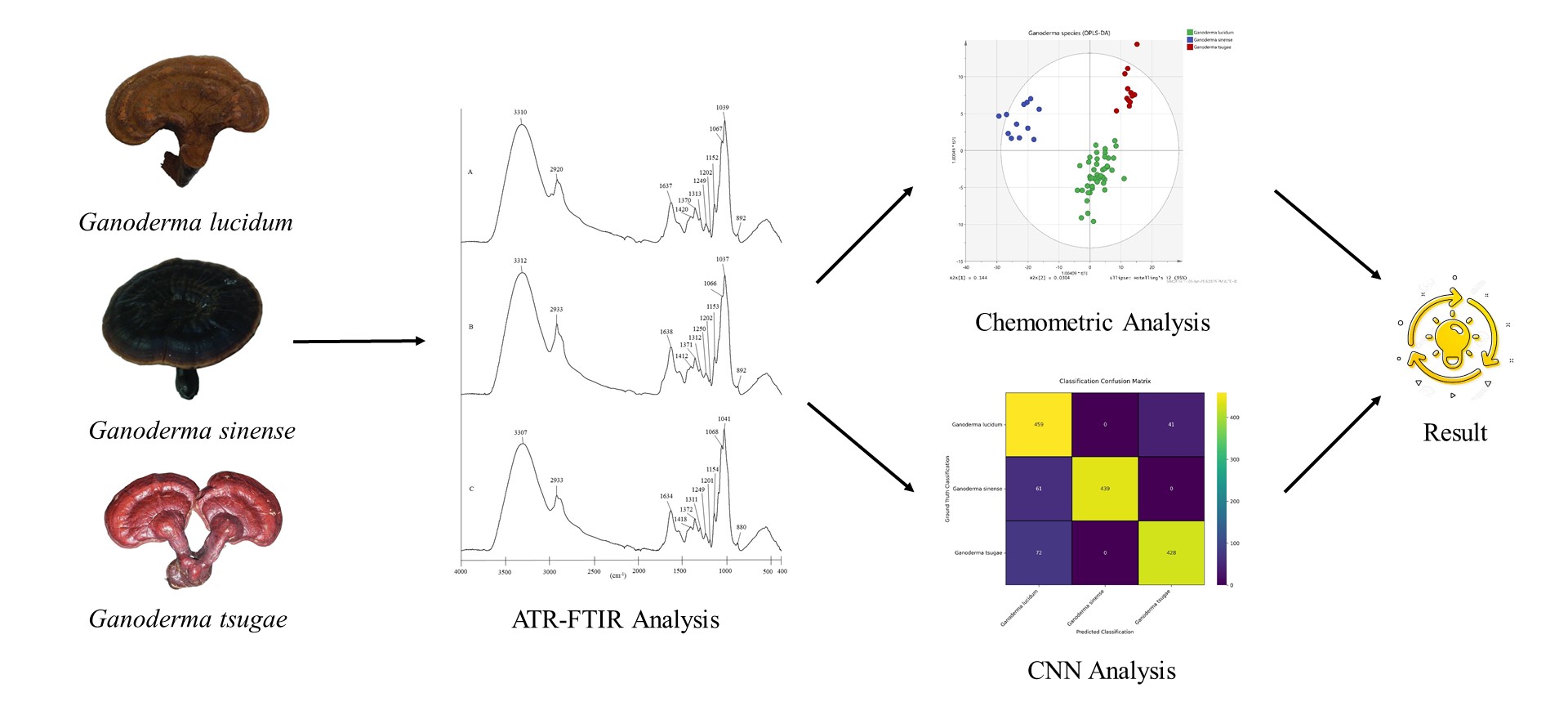

Supplement: Supplementary file 1 [file Image1.jpeg]
