# Supplementary material for: A rapid approach for discriminating Ganoderma species using attenuated total reflectance–Fourier transform infrared (ATR-FTIR) spectroscopy integrated with chemometric analysis and convolutional neural network (CNN)
Source: Front Chem. 2025 Oct 27;13:1655760. doi: 10.3389/fchem.2025.1655760 (PMC12597922; doi:10.3389/fchem.2025.1655760)
Supplement: Supplementary file 2 [file DataSheet1.docx]

The architecture of CNN

1. Convolutional layers

The model consists of two convolutional layers. The convolutional layers serve as the fundamental component and most essential unit of the CNN model. These convolutional layers consist of various filters which is also known as kernels. The filters or kernels will be gradually optimised throughout the training process. The size of filters was smaller than the images and is able to slid across the image. The dot product between the weights of the filters as well as the value of image data were estimated. This process will convert image data into an activation map or feature map which highlights the most significant features fed into the model including edges, textures, objects, and scenes. These activation maps are important as they were later applied for the purpose of classification. Owing to the input of one-dimensional ATR-FTIR spectral data in this model, one-dimensional convolutional arrays were applied in our model instead of the standard two-dimensional convolutional layers which is deemed inappropriate in this case.

2. Activation function

In a neural network model, activation function (AF) works to select essential and significant information from the data fed into the model while suppressing the irrelevant data. Thus, the model will be able to achieve task-specific goals despite reducing feature redundancy effectively (Khagi & Kwon, 2022). In both of the convolutional layers in our model, the well-known activation function, Rectified Linear Unit (ReLU) were employed. Despite the active development of various types of activation functions in the recent years, their inconsistent performance has meant that ReLU remains the standard (Bingham & Miikkulainen, 2022; Maas et al., 2013). While some of these new functions may show significant improvements compared to ReLU, however, ReLU is still regarded as more reliable activation function (Nwankpa et al., 2018) . Aside from having consistent and reliable performance, ReLU is well known in its ability to overcome vanishing gradient as compared to tanh and Sigmoid activation function. Also, ReLU contributes to rapid training times (Bingham & Miikkulainen, 2022).

3. Maxpooling layer

Pooling layers is included in a CNN model to aggregate and summarise features generated from convolutional layer. This enables down sampling and parameters reduction which minimise computational complexity (Nirthika et al., 2022). The effect of slight translation is also resisted by the pooling layers (Wang et al., 2018). The type of pooling layer applied in this model was max pooling layer. Max pooling layers surpass the performance of other pooling layer including average pooling and stochastic pooling as proven in several recent studies (Wang et al., 2018; Yu et al., 2015).

4. Regularisation – Early stopping & Batch normalisation

Both early stopping and batch normalisation were implemented in CNN model to address one of the most common issues in multi-layered machine learning which is overfitting. These measures taken to rectify the issue of overfitting are referred as regularisation, which adds information to control the model complexity by preventing excessive focus on the irrelevant features or noises (Friedrich et al., 2023). To elaborate further, the overly specialised model would be too sensitive and fit too closely towards noise in training set and fail to generalise the future data (Xu et al., 2023). This weakened generalisation will influence the accuracy and performance of the predictive model (Xu et al., 2023). Early stopping approach employed in CNN model was also applied by many recent statistical learning approaches. Plainly, overfitting means to stop the fitting process during the multiple iterations of validation dataset training before the model becomes more complex and leads to the occurrence of overfitting (Friedrich et al., 2023). The training process were stopped based on predefined criteria. We are also able to monitor the performance of the model during this approach. Hence, with this approach, its effective to retain the high model accuracy without losing its good generalisability. The second regularisation technique is batch normalisation. Recent studies have shown a trend of this technique effectively replacing another popular regularisation technique, the dropout layers in neural network model architecture (Garbin et al., 2020; Ioffe & Szegedy, 2015). Batch normalisation reduces the number of iterations as it normalises the output of every layer at each iteration. By minimising the number of iterations needed in a model is able to mitigate overfitting issue, retaining comparable accuracy with a faster computation process. The early employment of batch normalisation at the optimisation stage of a CNN model is encouraged as it can improve the model accuracy by 2 to 3% without leading to a significant structural changes on the model architecture (Garbin et al., 2020). In our study, batch normalisation outperformed dropout layers as a significant deterioration in the performance of the model was detected during the stage of evaluation. Thus, batch normalisation technique was adopted for regularisation purpose in our study.

5. Flatten layer

Flatten layer is one of the components CNN model architecture of this study. Flatten layer was applied to convert or flatten the multi-dimensional activation map into one-dimensional array. This process is fundamental to enable the understanding, learning and interpretation for the data classification by the fully connected dense layer.

6. Softmax layer

Another component of CNN model is the softmax layer. Typically, softmax layer is designed as the final layer of a classification CNN model. It functions to standardise the output of each classes into vectors of probability values whereby the sum of all vectors equate to 1 (Gao et al., 2020). This normalised distribution of the CNN output would lead to a smooth classification by the model.

**Reference**

Bingham, G., & Miikkulainen, R. (2022). Discovering Parametric Activation Functions. *Neural Networks*, *148*, 48-65. <https://doi.org/https://doi.org/10.1016/j.neunet.2022.01.001>

Friedrich, S., Groll, A., Ickstadt, K., Kneib, T., Pauly, M., Rahnenführer, J., & Friede, T. (2023). Regularization approaches in clinical biostatistics: A review of methods and their applications. *Statistical Methods in Medical Research*, *32*(2), 425-440. <https://doi.org/10.1177/09622802221133557>

Gao, Y., Liu, W., & Lombardi, F. (2020, 12-14 Oct 2020). Design and Implementation of an Approximate Softmax Layer for Deep Neural Networks. 2020 IEEE International Symposium on Circuits and Systems (ISCAS), Seville, Spain.

Garbin, C., Zhu, X., & Marques, O. (2020). Dropout vs. batch normalization: an empirical study of their impact to deep learning. *Multimedia Tools and Applications*, *79*(19), 12777-12815. <https://doi.org/10.1007/s11042-019-08453-9>

Ioffe, S., & Szegedy, C. (2015). Batch Normalization: Accelerating Deep Network Training by Reducing Internal Covariate Shift. *ArXiv*, *abs/1502.03167*. <https://doi.org/10.48550/arXiv.1502.03167>

Khagi, B., & Kwon, G.-R. (2022). A novel scaled-gamma-tanh (SGT) activation function in 3D CNN applied for MRI classification. *Scientific Reports*, *12*(1), 14978. <https://doi.org/10.1038/s41598-022-19020-y>

Maas, A. L., Hannun, A. Y., & Ng, A. Y. (2013). Rectifier nonlinearities improve neural network acoustic models. Proc. icml, Atlanta, GA.

Nirthika, R., Manivannan, S., Ramanan, A., & Wang, R. (2022). Pooling in convolutional neural networks for medical image analysis: a survey and an empirical study. *Neural Computing and Applications*, *34*(7), 5321-5347. <https://doi.org/10.1007/s00521-022-06953-8>

Nwankpa, C., Ijomah, W. L., Gachagan, A., & Marshall, S. (2018). Activation Functions: Comparison of trends in Practice and Research for Deep Learning. *ArXiv*, *abs/1811.03378*. <https://doi.org/10.48550/arXiv.1811.03378>

Wang, S.-H., Phillips, P., Sui, Y., Liu, B., Yang, M., & Cheng, H. (2018). Classification of Alzheimer’s Disease Based on Eight-Layer Convolutional Neural Network with Leaky Rectified Linear Unit and Max Pooling. *Journal of Medical Systems*, *42*(5), 85. <https://doi.org/10.1007/s10916-018-0932-7>

Xu, C., Coen-Pirani, P., & Jiang, X. (2023). Empirical Study of Overfitting in Deep Learning for Predicting Breast Cancer Metastasis. *Cancers*, *15*(7), 1969. <https://www.mdpi.com/2072-6694/15/7/1969>

Yu, X., Yang, J., Wang, T., & Huang, T. (2015). Key Point Detection by Max Pooling for Tracking. *IEEE Transactions on Cybernetics*, *45*(3), 430-438. <https://doi.org/10.1109/TCYB.2014.2327246>
